# Beyond 25(OH)D: Carboxylated Osteocalcin and the Undercarboxylated/Carboxylated Osteocalcin Ratio as Superior Biomarkers for Vitamin D Recovery in Offspring Affected by Maternal Deficiency

**DOI:** 10.3390/nu18081243

**Published:** 2026-04-15

**Authors:** Wai-Tao Chan, Hung-Chang Lee, Chun-Yan Yeung, Jen-Shiu Chiang Chiau, Mei-Lein Cheng, Szu-Wen Chang, Shu-Chao Weng, Chuen-Bin Jiang

**Affiliations:** 1 Department of Pediatric Gastroenterology, Hepatology and Nutrition, MacKay Children’s Hospital, Taipei 104217, Taiwan; taody@mmh.org.tw (W.-T.C.); 8231boss@gmail.com (H.-C.L.); cyyeung1029@gmail.com (C.-Y.Y.); changszuwen@gmail.com (S.-W.C.); shuchao@gmail.com (S.-C.W.); 2 Department of Medical Research, MacKay Memorial Hospital, Taipei 104217, Taiwan; chiang1997@hotmail.com (J.-S.C.C.); dafene_c@hotmail.com (M.-L.C.); 3 Department of Medicine, School of Medicine, MacKay Medical University, New Taipei City 252005, Taiwan; 4 MacKay Junior College of Medicine, Nursing, and Management, New Taipei City 252005, Taiwan

**Keywords:** bone matrix, carboxylated osteocalcin, maternal vitamin D deficiency, offspring, osteocalcin

## Abstract

**Background**: Maternal vitamin D deficiency (VDD) compromises fetal skeletal development. The impact of postnatal vitamin D supplementation on osteocalcin (OC) carboxylation, converting undercarboxylated (ucOC) to carboxylated osteocalcin (cOC), in offspring remains unclear, given conflicting reports on the correlation between serum 25-hydroxyvitamin D (25(OH)D) and specific OC forms. This study investigated OC profile recovery in a mouse model of maternal VDD. **Methods**: Female C57BL/6J mice were fed a VDD diet from four weeks pre-conception through lactation. Weaned offspring were maintained on the VDD diet and randomized to three groups: control (saline), standard-dose (1500 IU/kg), or high-dose (4500 IU/kg) vitamin D supplementation. Serum 25(OH)D, cOC, and ucOC were quantified via ELISA at 1, 2, and 4 weeks post-intervention. **Results**: Controls remained vitamin D-deficient (<13 ng/mL). Supplementation dose-dependently increased serum 25(OH)D (*p* < 0.05). Crucially, while absolute ucOC levels remained stable across all groups, supplementation significantly upregulated cOC and total osteocalcin at all time points (*p* < 0.05). Consequently, the ucOC/cOC ratio significantly decreased in supplemented groups. Partial correlation analysis revealed a strong positive correlation between 25(OH)D and cOC (r_partial_ = 0.718) and a negative correlation with the ucOC/cOC ratio (r_partial_ = −0.433), but no correlation with ucOC (r_partial_ = −0.102). **Conclusions**: In offspring affected by maternal VDD, vitamin D supplementation improves the osteocalcin carboxylation profile primarily by driving carboxylated osteocalcin synthesis rather than reducing the undercarboxylated pool. Serum cOC and the ucOC/cOC ratio serve as superior functional biomarkers to ucOC for monitoring therapeutic efficacy in this early-life developmental model.

## 1. Introduction

Maternal nutrition during pregnancy is a fundamental determinant of fetal development and lifelong metabolic health. Among essential micronutrients, maternal vitamin D deficiency (VDD) remains a pervasive global health issue, profoundly compromising fetal skeletal mineralization. The human fetus relies entirely on the placental transfer of 25-hydroxyvitamin D (25(OH)D); consequently, inadequate maternal dietary intake or low sun exposure inevitably leads to a depleted fetal vitamin D pool and congenital osteopenia [1,2]. While the role of vitamin D in calcium homeostasis is well established, its regulatory effect on the functional quality of the bone matrix—specifically through its interaction with other fat-soluble vitamins—requires further elucidation.

A critical downstream target of the active vitamin D metabolite, 1,25-dihydroxyvitamin D3 (1,25(OH)2D3), is osteocalcin (OC), the most abundant non-collagenous protein in the bone matrix [3]. Vitamin D strongly upregulates the transcription of the osteocalcin gene *BGLAP* in osteoblasts [4,5]. However, the functional competency of this newly synthesized protein relies heavily on a synergistic nutritional interplay with vitamin K [6]. Vitamin K acts as an essential cofactor for γ-glutamyl carboxylase, an enzyme responsible for the post-translational carboxylation of glutamate residues on the osteocalcin molecule [3,7]. This modification converts undercarboxylated osteocalcin (ucOC) into carboxylated osteocalcin (cOC), which exhibits a high affinity for hydroxyapatite and facilitates bone matrix maturation [8,9]. Thus, optimal bone formation requires a coordinated nutritional axis: vitamin D to drive protein synthesis and vitamin K to ensure its functional activation.

Despite this established biochemical synergy, clinical evidence regarding the correlation between serum 25(OH)D levels and specific osteocalcin carboxylation profiles is surprisingly inconsistent. Torbergsen et al. reported a positive correlation between 25(OH)D and total osteocalcin in elderly populations [10], while Bunyaratavej et al. observed a positive correlation specifically with ucOC when 25(OH)D levels were repleted [11]. In contrast, Szulc et al. found a negative correlation between ucOC and vitamin D, suggesting that correcting VDD might play a role in normalizing osteocalcin carboxylation [12]. Furthermore, recent nutritional studies highlight the clinical relevance of the ucOC/cOC ratio as a sensitive biomarker for bone fragility and vitamin K status [13,14].

These discrepancies likely stem from variations in baseline nutritional status and the age of the studied populations. Crucially, most prior studies have focused on adults or the elderly, where bone resorption dominates. There is a profound lack of data regarding this nutrient interaction in the context of early-life nutritional programming and maternal VDD. It remains unclear whether targeted postnatal dietary intervention (vitamin D supplementation) in neonates merely restores systemic 25(OH)D levels or effectively rescues the functional quality of the bone matrix by modulating the carboxylation profile.

Crucially, addressing this knowledge gap in human pediatric cohorts presents significant ethical and methodological constraints. It is ethically prohibitive to conduct controlled, untreated VDD longitudinal studies or perform frequent, large-volume blood sampling in human neonates to capture early biochemical kinetics. Furthermore, clinical cohorts are inevitably confounded by diverse maternal diets, variable prenatal vitamin K status, and heterogeneous postnatal sun exposure. Therefore, a strictly controlled murine model of maternal VDD is essential to isolate the precise temporal and mechanistic impact of postnatal vitamin D rescue on the osteocalcin carboxylation profile without these confounding factors.

In this study, we utilized a murine model of maternal VDD to bridge this knowledge gap. We hypothesized that in offspring metabolically compromised by maternal VDD, post-weaning vitamin D supplementation would act synergistically with basal dietary vitamin K to enhance serum cOC levels and improve the carboxylation ratio (ucOC/cOC), thereby restoring bone matrix homeostasis.

## 2. Materials and Methods

### 2.1. Ethics Statement and Animal Maintenance

This study was conducted in strict accordance with the Animal Protection Act of Taiwan and the Guideline for the Care and Use of Laboratory Animals (Council of Agriculture, Executive Yuan, Taiwan). The experimental protocol was permitted by the Institutional Animal Care and Use Committee (IACUC) of MacKay Memorial Hospital (Approval Number: MMH-A-S-111-14). C57BL/6J mice were obtained from the National Center for Biomodels (NCB, Taipei, Taiwan). All animals were housed in a specific pathogen-free (SPF) facility under controlled environmental conditions, including a 12-h light/dark cycle and constant temperature (20–25 °C), with ad libitum access to diet and water.

### 2.2. Generation of Maternal Vitamin D Deficiency Model

A mouse model of maternal vitamin D deficiency (VDD) was established following the protocol previously described by Yeung et al. [15]. Female C57BL/6J mice (8–10 weeks old) were fed a customized vitamin D-deficient diet for inducing hypovitaminosis D in mice (Product No. E15312-24, ssniff Spezialdiäten GmbH, Soest, Germany) for four weeks prior to mating to deplete systemic stores. Notably, no vitamin K supplements were added to the feeds as the customized diet contained menadione nicotinamide bisulfate 20 mg/kg itself. The diet was maintained throughout gestation and lactation to keep maternal VDD before weaning of the pups. For those offspring, the successful induction and persistence of vitamin D deficiency would be confirmed by longitudinal measurements of serum 25(OH)D levels in the unsupplemented offspring.

### 2.3. Post-Weaning Supplementation and Experimental Grouping

Upon weaning, male offspring were selected from dams and maintained on the VDD diet. Pups were randomized into three intervention groups using a computer-generated random number sequence, ensuring that offspring from the same litter were evenly distributed across different treatment arms to minimize litter-specific bias. They received weekly treatment via oral gavage using cholecalciferol (Vitamin D3, 5000 IU/tablet, General Nutrition Corporation, Pittsburgh, PA, USA). Because vitamin D is highly lipophilic and insoluble in aqueous media, we established a standardized transient suspension protocol for precise low-volume oral gavage. The tablets were finely pulverized into a homogeneous powder. Immediately prior to each feeding session, the required dose of the pulverized vitamin D was added to a normal saline vehicle. The mixture was subjected to vigorous vortexing to create a uniform, transient aqueous suspension and was instantaneously administered to the offspring via oral gavage. This rapid dispensing process ensured consistent and accurate dose delivery before any particulate settling or phase separation could occur.

1. Control group (C): Received saline vehicle, no vitamin D supplementation.
2. Standard-dose group (S): Supplemented with 1500 IU/kg vitamin D. This dosage was selected to mirror the standard physiological maintenance requirement typical of standard rodent chow (Laboratory Autoclavable Rodent Diet 5010; LabDiet, St. Louis, MO, USA).
3. High-dose group (H): Supplemented with 4500 IU/kg vitamin D. This 3-fold higher dosage was established to represent a therapeutic intervention designed to rapidly rescue depleted stores without inducing hypervitaminosis.

To assess the temporal effects of supplementation, animals from each dosage group were sacrificed at three distinct time points: 1, 2, and 4 weeks post-intervention. This resulted in a total of nine experimental subgroups (e.g., C-1W, S-1W, H-1W), utilizing a total of 45 offspring mice, with 5 animals randomly assigned to each subgroup. All procedures, including blood sampling, were performed under isoflurane anesthesia to minimize animal suffering. At the designated endpoints, mice were euthanized by via cardiac puncture then cervical dislocation under deep isoflurane anesthesia.

### 2.4. Analysis

Serum samples were collected at the designated endpoints. Serum 25-hydroxyvitamin D (25(OH)D) concentrations were quantified using a commercial ELISA kit (EAGLE Bioscience, Inc., Amherst, NH, USA). Osteocalcin profiles were assessed using specific ELISA kits for undercarboxylated osteocalcin (ucOC) and carboxylated osteocalcin (cOC) (MyBioSource, Inc., San Diego, CA, USA), following the manufacturer’s instructions. Total osteocalcin (tOC) was calculated as the sum of ucOC and cOC concentrations. To minimize bias, all biochemical assays (ELISA) and subsequent statistical analyses were performed by investigators who were blinded to the animal group allocations. Because these two fractions represent the entirety of the circulating osteocalcin pool, mathematically adding their absolute concentrations provides a reliable measure of total protein synthesis without cross-reactivity issues, an approach previously validated [16].

### 2.5. Statistical Analysis

Data management and analysis were performed using SPSS Statistics 21 (IBM Corp., Armonk, NY, USA). Quantitative data are presented as mean ± standard deviation (SD). To prevent Type I error inflation arising from multiple comparisons across the 3 × 3 experimental design, statistical analyses were comprehensively conducted using the two-way analysis of variance (ANOVA). This approach evaluated the main effects of the intervention (dose) and duration (time), alongside their interaction. When significant main effects or interactions were detected, Tukey’s post hoc test was applied for appropriate multiple pairwise comparisons. Relationships between serum 25(OH)D levels and osteocalcin parameters (tOC, cOC, ucOC, and the ucOC/cOC ratio) were assessed using partial correlation analysis presenting with the corresponding coefficient r_partial_. A *p* value < 0.05 was considered statistically significant.

## 3. Results

The blood levels of serum 25(OH)D and osteocalcin parameters across all time points were detailed in Table 1.

For the 3 × 3 experimental design in our study, two-way analysis of variance (ANOVA) was applied for statistical analysis to verify significance. The main effects of the intervention (dose) and duration (time), alongside their interaction, were checked, as shown in Table 2. When significant main effects or interactions were detected, Tukey’s post hoc test was applied for appropriate multiple pairwise comparisons.

### 3.1. Restoration of Serum Vitamin D Levels via Supplementation

**Figure 1:**
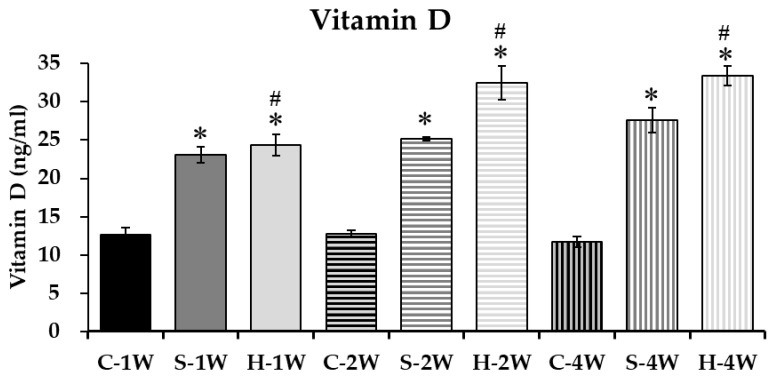
Serum vitamin D levels in offspring of VDD dams after 1-, 2- & 4-week intervention. Values are presented as mean ± SD. Statistical significance was determined by Two-way ANOVA followed by Tukey’s post hoc multiple comparisons test. * *p* < 0.05 compared to the control group at the same time point; ^#^ *p* < 0.05 compared to the standard-dose group at the same time point.

The maternal VDD protocol successfully established a persistent deficiency in the offspring. Offspring in the control group (C) maintained serum 25(OH)D levels in the deficient range (<13 ng/mL) throughout the entire experimental period (Table 1). Post-weaning vitamin D supplementation effectively reversed this state. As shown in Figure 1, serum 25(OH)D levels significantly increased in both the standard- (S) and high-dose (H) groups compared to controls at all time points (*p* < 0.05). Notably, a dose-dependent response was observed; mice supplemented with the high dose (4500 IU/kg) exhibited significantly higher serum 25(OH)D concentrations compared to those receiving the standard dose (1500 IU/kg).

### 3.2. Effect of Supplementation on Carboxylated Osteocalcin (cOC)

**Figure 2:**
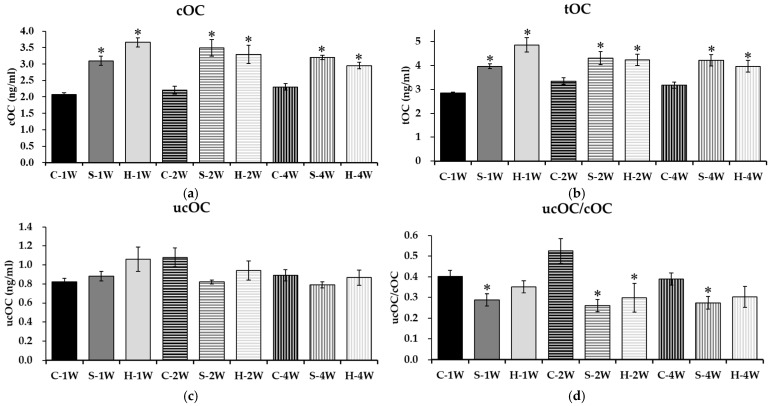
Impact of vitamin D supplementation on osteocalcin carboxylation profiles. (**a**) Restoration of serum carboxylated osteocalcin (cOC) levels; (**b**) total osteocalcin (tOC) production; (**c**) stability of serum undercarboxylated osteocalcin (ucOC) concentrations; (**d**) improvement in osteocalcin carboxylation efficiency as indicated by the ucOC/cOC ratio. Data are presented as mean ± SD. Statistical significance was determined by Two-way ANOVA followed by Tukey’s post hoc multiple comparisons test. * *p* < 0.05 compared to the control group at the same time point.

To evaluate the impact of vitamin D on bone matrix maturation, we measured serum cOC levels (Figure 2a). In the unsupplemented control group, cOC levels showed no significant changes at 1, 2, or 4 weeks. However, vitamin D supplementation induced a marked upregulation of cOC. Offspring in both the standard- (S) and high-dose (H) groups exhibited statistically significant increases in serum cOC concentrations compared to controls at all observed time points (1, 2, and 4 weeks; *p* < 0.05), indicating active synthesis of the mature protein form.

### 3.3. Changes in Total Osteocalcin (tOC)

Consistent with the rise in cOC, tOC levels—representing the sum of carboxylated and undercarboxylated forms—remained low in the unsupplemented control group but increased significantly following intervention (Figure 2b). Both supplementation dosages (S and H) resulted in significantly elevated tOC levels at the end of the first, second, and fourth weeks compared to controls (*p* < 0.05), reflecting enhanced overall osteoblast activity.

### 3.4. Stability of Undercarboxylated Osteocalcin (ucOC)

In contrast to cOC and tOC, serum ucOC levels exhibited variation but showed no consistent pattern of change related to vitamin D status (Figure 2c). Statistical analysis revealed no significant differences in ucOC concentrations between the unsupplemented controls and the supplemented groups (S or H) at any time point. This suggests that while vitamin D drives total osteocalcin production, it does not significantly alter the absolute pool of the undercarboxylated fraction in this model.

### 3.5. Improvement in Carboxylation Efficiency (ucOC/cOC Ratio)

The ratio of ucOC to cOC (ucOC/cOC), a sensitive indicator of vitamin K-dependent carboxylation efficiency, demonstrated significant improvement with supplementation (Figure 2d). In the unsupplemented control group, the ratio remained unchanged. In the standard-dose group (S), the ucOC/cOC ratio was significantly lower than that of controls at weeks 1, 2, and 4 (*p* < 0.05). In the high-dose group (H), a significant reduction in the ratio was specifically observed at the second week of treatment (*p* < 0.05).

### 3.6. Relationships Between Serum 25(OH)D Levels and Osteocalcin Parameters

To ensure that the associations between vitamin D and ucOC, cOC, and tOC, as well as ucOC/cOC, were not predominantly driven by between-group treatment effects (Simpson’s paradox), we subsequently performed partial correlation analyses controlling for treatment group and time point. The positive association between 25(OH)D and cOC (r_partial_ = 0.718, *p* < 0.001) and the negative association with the ucOC/cOC ratio (r_partial_ = −0.433, *p* = 0.006) persisted significantly, confirming a genuine intra-physiological relationship, as shown in Table 3.

## 4. Discussion

### 4.1. Summary of Main Findings

This study provides novel insights into the nutritional rescue of bone matrix markers in offspring affected by maternal vitamin D deficiency (VDD). Utilizing a validated maternal VDD mouse model, we demonstrated that targeted post-weaning dietary intervention (vitamin D supplementation) acts as a potent driver for the synthesis of carboxylated osteocalcin (cOC) and total osteocalcin (tOC). Remarkably, this intervention left the absolute pool of undercarboxylated osteocalcin (ucOC) largely unchanged. Consequently, vitamin D supplementation effectively reduced the ucOC/cOC ratio, shifting the biochemical profile towards a more mature, carboxylated state. Interestingly, our post hoc analyses revealed no significant differences in the magnitude of cOC elevation between the standard- and high-dose groups across the time points. This observation strongly implies a biological ‘ceiling effect’ or plateau in osteocalcin carboxylation. It suggests that the standard dose (1500 IU/kg) is already sufficient to saturate either the transcriptional capacity of the osteoblasts or the enzymatic limits of the r-glutamyl carboxylase machinery in this early-life model.

### 4.2. Maternal Nutritional Programming and Postnatal Rescue

The skeletal health of the neonate is deeply programmed by the maternal nutritional environment. Consistent with the Developmental Origins of Health and Disease (DOHaD) paradigm, our model confirms that inadequate maternal vitamin D status strictly limits the fetal pool, resulting in profound post-weaning deficiency (<13 ng/mL) in the offspring [17,18]. Our findings highlight the efficacy of timely postnatal nutritional intervention; supplementation rapidly and dose-dependently restored systemic 25(OH)D levels, reversing the trajectory of early-life deficiency.

### 4.3. Mechanistic Insights: The Vitamin D and Vitamin K Synergy

The most striking observation in our study was the selective upregulation of cOC and the stability of ucOC levels following vitamin D intervention. This specific pattern highlights a critical micronutrient crosstalk between vitamin D and vitamin K. Mechanistically, the active metabolite 1,25(OH)2D3 binds to the vitamin D receptor (VDR) to directly enhance the transcription of the *BGLAP* gene, initiating a surge in de novo osteocalcin synthesis [19,20].

We hypothesize that in our rapidly growing offspring model, vitamin D serves as the rate-limiting factor for bone matrix protein quantity. The observation that newly synthesized osteocalcin was efficiently converted to cOC suggests the possibility that the endogenous vitamin K status was sufficient. However, as serum vitamin K levels were not directly quantified, this mechanistic interpretation remains a hypothesis. Follow-up experiments incorporating strictly modulated dietary vitamin K and direct quantification of serum phylloquinone/menaquinone are required to definitively test the limits of this proposed vitamin D–vitamin K synergy. In this context, the two fat-soluble vitamins operate in perfect synergy: dietary vitamin D unlocks the production of the protein substrate, while the existing vitamin K pool ensures its functional maturation (carboxylation). This explains why the ucOC pool remained stable; the carboxylation machinery was not overwhelmed, preventing the spillover of immature ucOC into the circulation [21,22].

The absence of a consistently significant reduction in the ucOC/cOC ratio in the high-dose group at weeks 1 and 4, despite significant improvements in the standard-dose group, warrants attention. This may reflect a non-linear, temporal dose–response relationship. We hypothesize that a sudden, high-dose pharmacological bolus of vitamin D may trigger a rapid burst of de novo osteocalcin synthesis that transiently outpaces the endogenous capacity of the vitamin K-dependent carboxylation machinery, resulting in temporary fluctuations in the ratio.

### 4.4. Resolving the Biomarker Controversy

This nutritional synergy helps resolve conflicting reports in the literature. While some studies in elderly osteoporotic populations reported positive [11] or negative [23] correlations between vitamin D and ucOC, those models often involve age-related declines in vitamin K absorption or altered bone turnover kinetics. In contrast, our early-life model represents a highly anabolic state. Our data strongly suggest that in the context of developmental VDD recovery, the lack of change in ucOC does not imply an ineffective nutritional intervention. Instead, the robust increase in cOC and the suppression of the ucOC/cOC ratio serve as definitive proof of restored bone matrix quality.

### 4.5. Nutritional Implications and Limitations

From a clinical nutrition perspective, these findings advocate for a paradigm shift in how we monitor therapeutic efficacy in pediatric VDD. The ucOC/cOC ratio emerged as a highly sensitive functional biomarker of the vitamin D-K axis, outperforming ucOC alone. Clinicians and dietitians should consider this ratio when evaluating the success of dietary interventions in high-risk infants.

### 4.6. Translational Relevance and Clinical Application

In real-world clinical practice, monitoring the recovery of pediatric VDD relies almost exclusively on achieving serum 25(OH)D levels > 30 ng/mL. However, this static measure does not confirm functional bone matrix maturation. Our findings suggest that translating the ucOC/cOC ratio into clinical assessment could offer several advantages. For instance, in treating infants born to VDD mothers or premature neonates at risk for metabolic bone disease, clinicians could monitor cOC trajectories to confirm therapeutic efficacy at the tissue level. Furthermore, a persistently high ucOC/cOC ratio despite normalized 25(OH)D levels could alert pediatricians to a secondary, hidden vitamin K bottleneck, guiding targeted co-supplementation rather than aggressively increasing vitamin D dosages, thereby mitigating the risk of hypercalcemia. While these applications require robust validation in prospective pediatric cohorts, they represent a significant step toward precision nutritional monitoring.

### 4.7. Limitations

We acknowledge certain limitations in our study. First, as accurately pointed out, we did not directly quantify serum phylloquinone (vitamin K1) or menaquinone (vitamin K2) levels. Due to the technical constraints associated with the extremely limited blood volume in neonatal and young mice, prioritizing assays was necessary. Second, an a priori statistical power calculation was not performed; the sample size was based on established precedents for similar nutritional models to adhere to the 3Rs principles of animal welfare. Consequently, the study may be underpowered to detect subtle or moderate changes in certain highly variable markers, such as absolute ucOC levels. Third, our biochemical analysis was strictly focused on the osteocalcin carboxylation profile. Due to the severely limited serum volumes obtainable from weaning mice, we were unable to simultaneously quantify other standard markers of bone remodeling, such as bone-specific alkaline phosphatase (BALP) or TRACP-5b. Consequently, while our data confirms the maturation of the osteocalcin matrix pool, the broader holistic dynamics of osteoblast and osteoclast activity during this rapid recovery phase remain to be fully elucidated in future studies. Fourth, a major limitation of this study is the absence of direct morphological or histological assessments of the skeleton. Because our primary experimental endpoint was the temporal biochemical characterization of the circulating osteocalcin profile, we did not perform micro-computed tomography (micro-CT) or bone histomorphometry. Therefore, while the significant reduction in the ucOC/cOC ratio strongly indicates an improvement in biochemical bone matrix markers, direct physical improvements in trabecular microarchitecture or cortical bone strength cannot be definitively claimed. Future investigations incorporating detailed structural and biomechanical analyses are essential to directly correlate these circulating biomarker shifts with actual skeletal integrity and physical bone improvement.

Therefore, we relied on the ucOC/cOC ratio as a surrogate marker of functional vitamin K status in the bone. While our data highlight the robust upregulation of cOC, the stable yet variable profile of ucOC must be interpreted with caution. Given the relatively small sample size, our study may be underpowered to detect moderate, transient changes in the absolute ucOC pool. Future investigations utilizing larger cohorts or pooled longitudinal data are necessary to definitively confirm the absolute stability of ucOC under these conditions. Finally, the translational limitations of extrapolating these findings from a murine model to human pediatric practice must be explicitly acknowledged. Mice exhibit vastly accelerated bone modeling kinetics and different metabolic turnover rates compared to human infants. Before the ucOC/cOC ratio can be recommended for routine clinical use, robust clinical validation is essential. Future research must involve prospective, longitudinal pediatric cohorts to examine how changes in the ucOC/cOC ratio correlate with definitive clinical outcomes, such as dual-energy X-ray absorptiometry (DXA)-derived bone mineral density (BMD), long-term fracture risk, and skeletal growth velocity during early childhood. Moreover, future nutritional studies should concurrently measure both vitamins to fully map their kinetic interactions. Correlating these biochemical shifts with direct micro-computed tomography structural analyses will further solidify the structural impact of this nutritional synergy

## 5. Conclusions

In conclusion, targeted post-weaning vitamin D supplementation effectively rescues the bone matrix profile of offspring compromised by maternal VDD. By driving the synthesis of carboxylated osteocalcin in synergy with basal vitamin K, this dietary intervention significantly improves the carboxylation ratio. These findings emphasize the importance of micronutrient interactions in early development and highlight cOC and the ucOC/cOC ratio as superior functional biomarkers for monitoring nutritional recovery in pediatric populations.

## Figures and Tables

**Table 1:** Absolute mean values of serum 25-hydroxyvitamin D and osteocalcin carboxylation profiles across different intervention groups and time points.

Parameter	Time Point	Control	Standard-Dose	High-Dose
25(OH)D (ng/mL)	week 1	12.7 ± 1.9	23.1 ± 2.3 *	24.4 ± 3.1 *^#^
	week 2	12.7 ± 1.0	25.2 ± 0.6 *	32.5 ± 4.5 *^#^
	week 4	11.7 ± 1.6	27.6 ± 3.7 *	33.4 ± 2.9 *^#^
cOC (ng/mL)	week 1	2.1 ± 0.1	3.1 ± 0.3 *	3.7 ± 0.3 *
	week 2	2.2 ± 0.3	3.5 ± 0.5 *	3.5 ± 0.7 *
	week 4	2.3 ± 0.2	3.2 ± 0.1 *	3.0 ± 0.2 *
tOC (ng/mL)	week 1	2.9 ± 0.1	4.0 ± 0.2 *	4.9 ± 0.7 *
	week 2	3.4 ± 0.3	4.3 ± 0.5 *	4.3 ± 0.4 *
	week 4	3.2 ± 0.3	4.2 ± 0.5 *	4.0 ± 0.6 *
ucOC (ng/mL)	week 1	0.8 ± 0.1	0.9 ± 0.1	1.1 ± 0.3
	week 2	1.1 ± 0.2	0.8 ± 0.1	0.9 ± 0.2
	week 4	0.9 ± 0.1	0.8 ± 0.1	0.9 ± 0.2
ucOC/cOC ratio	week 1	0.4 ± 0.1	0.3 ± 0.1 *	0.4 ± 0.1
	week 2	0.5 ± 0.1	0.3 ± 0.1 *	0.2 ± 0.2 *
	week 4	0.4 ± 0.1	0.3 ± 0.1 *	0.4 ± 0.1

Data are presented as mean ± standard deviation (SD). Statistical significance was determined by Two-Way ANOVA followed by Tukey’s post hoc multiple comparisons test (Table 2). * *p* < 0.05 compared to the control group at the same time point. ^#^ *p* < 0.05 compared to the standard-dose group at the same time point. 25(OH)D, 25-hydroxyvitamin D; cOC, carboxylated osteocalcin; tOC, total osteocalcin; ucOC, undercarboxylated osteocalcin.

**Table 2:** Two-way ANOVA assessment of the main effects of time, dose, and their interaction on serum parameters in offspring mice.

Target	*p* Value		
	Time	Dose	Interaction
Vitamin D	<0.001	<0.001	0.001
cOC	0.176	<0.001	0.043
tOC	0.159	0.093	0.054
ucOC	0.521	<0.001	0.016
ucOC/cOC	0.957	<0.001	0.053

cOC, carboxylated osteocalcin; tOC, total osteocalcin; ucOC, undercarboxylated osteocalcin.

**Table 3:** The partial correlation test between vitamin D and ucOC, cOC, tOC as well as ucOC/cOC.

	Vitamin D			
	cOC	tOC	ucOC	ucOC/cOC
r_partial_	0.718	0.694	−0.102	−0.433
*p* value	<0.001	<0.001	0.544	0.006

cOC, carboxylated osteocalcin; tOC, total osteocalcin; ucOC, undercarboxylated osteocalcin.

## Data Availability

The original contributions presented in this study are included in the article. Further inquiries can be directed to the corresponding author.

## Abbreviations

The following abbreviations are used in this manuscript: VDD, vitamin D deficiency; 25(OH)D, 25-hydroxyvitamin D; 1,25(OH)2D3, 1,25-dihydroxyvitamin D3; OC, osteocalcin; ucOC, undercarboxylated osteocalcin; cOC, carboxylated osteocalcin; tOC, total osteocalcin; SPF, specific pathogen-free; SD, standard deviation; SPSS, Statistical Package for the Social Sciences; DOHaD, Developmental Origins of Health and Disease; VDR, vitamin D receptor.
